# Global Surveillance of Public Interest in Cosmetic Tourism for Aesthetic Eyelid Surgery Abroad: Cross-Sectional Infodemiology Investigation of Internet Search Trends and Social Media Content

**DOI:** 10.2196/64639

**Published:** 2025-06-02

**Authors:** Daniel B Azzam, Yi Ling Dai, Victoria S North, Alison B Callahan, Katrinka L Heher, Mitesh K Kapadia, M Reza Vagefi

**Affiliations:** 1 Division of Oculofacial Plastic & Orbital Surgery, New England Eye Center, Tufts Medicine, Biewend Building 11th Fl., 260 Tremont St, Boston, MA, 02116, United States, 1 617-636-4600, 1 617-636-4866

**Keywords:** cosmetic tourism, oculofacial plastic surgery, aesthetic eyelid surgery, Google Trends, social media, travel medicine, global health, infodemiology, digital epidemiology, Instagram, eyelids, aesthetic, medical tourism, eyelid surgery, plastic surgery, blepharoplasty

## Abstract

**Background:**

Global medical tourism for aesthetic surgery has become a popular phenomenon through ease of access in the digital era, though such services are not without potential risks. The application of infodemiology for global health surveillance may provide unique insights into unknown patient travel patterns and surgeon workforce dynamics abroad.

**Objective:**

This study aimed to evaluate American cosmetic tourism trends in oculofacial plastic surgery, including demand profile and qualifications of the most sought-after international eyelid surgeons on social media.

**Methods:**

This cross-sectional infodemiology study queried Google Trends to assess US interests in aesthetic eyelid surgery abroad in 25 destination countries from 2013 to 2023. The highest-rated content posted by 55 eyelid surgeons (US: n=11; international: n=44) on a social media platform (Instagram; Meta Platforms) was evaluated. The main outcomes included Google search volumes for aesthetic eyelid surgery for each destination country, as well as specialty training and professional medical society affiliations of popular eyelid surgeons on social media in each of these countries.

**Results:**

The top 5 destinations Americans sought for aesthetic eyelid surgery abroad were South Korea, Mexico, Canada, Turkey, and China. Interest in eyelid surgery abroad remained stable over the last decade despite 118% growth in blepharoplasty searches. Social media indicated eyelid surgeons abroad were more often general plastic surgeons than in the United States (30/44, 68% vs 2/11, 18%; *P*=.003). US surgeons more frequently completed oculofacial plastics, facial plastics, or aesthetic plastics fellowships compared with international surgeons (9/11, 82% vs 10/44, 23%; *P*<.001) and had membership in professional medical societies (11/11, 100% vs 22/44, 50%; *P*=.002).

**Conclusions:**

American demand for international eyelid surgery remained stable over the past decade despite a 2-fold increase in the US interest for blepharoplasty. Digital epidemiology data reveal a shortage of international surgeons with specialized aesthetic eyelid fellowship training or professional society affiliations on social media among the preferred destinations for Americans seeking aesthetic eyelid surgery. These findings may provide beneficial insights for patients interested in traveling abroad for eyelid surgery, as well as for surgeons or academic societies seeking to increase social media presence or patient-directed educational content via social media engagement.

## Introduction

The digital transformation driven by the internet and social media has reduced international boundaries in medical care, enabling aesthetic surgeons worldwide to market their services to patients abroad for cosmetic tourism [1].

Travel abroad for discounted cosmetic surgery may offer some putative benefits, including anonymity, access to procedures unavailable locally, and all-inclusive medical tourism packages. However, such services are not without potential risks or untoward consequences. Considerations include variable preoperative counseling, inadequate follow-up, inconsistent quality of care, language barrier, postoperative care burden on the home health care system, and financial and legal challenges in the event of complications [2,3]. Furthermore, centers abroad may not hold surgeons to the same training standards or safety regulations as the United States [4].

Eyelid surgery ranks third in aesthetic surgeries worldwide [5]. Google Trends (Alphabet Inc) is a valuable tool for health information–seeking behavior and has been employed to analyze aesthetic surgery demand within oculofacial plastic surgery (OPS), facial cosmetic surgery, facial feminization surgery, and cosmetic tourism for plastic surgery [3,6-8].

Social media is a dominant platform for medical advice, patient education, and business marketing in this field [9]. A study of nearly 400 survey participants showed that 49% (n=193) of patients found their oculofacial plastic surgeon on social media, with more than two-thirds choosing Instagram (Meta Platforms) as the preferred social media platform to find an oculofacial plastic surgeon [10].

Previous studies on social listening within OPS have largely focused on marketing strategies through content category analysis [11]. In this regard, Cheng et al [9] showed that the OPS content category amassing the most views was “live procedure or surgeries” followed by “educational” and “patient experience.” Park et al [12] demonstrated that OPS photographs were more successful than videos, carousel presentation was better than collage, and featuring the posting doctor, especially when smiling and wearing a white coat, increased public engagement. Similarly, other social media listing research in aesthetic plastic surgery and ophthalmology have demonstrated the success of various hashtag utilization in social media marketing and the prevalence of inaccurate medical information among patient-generated Reddit discussions [13,14].

This study fills an important gap in the social listening literature for eyelid surgery from the perspective of global health and, to the authors’ knowledge, is the first paper examining social media as a space for cosmetic tourism patients to find international OPS providers. The authors aimed to use a digital epidemiology approach to analyze current global health trends in OPS cosmetic tourism sought by Americans over the last decade. Furthermore, this study undertakes an analysis of social media data to compare training backgrounds and professional academic society affiliations of social media’s most popular international eyelid surgeons. These findings may provide beneficial insights for patients interested in traveling abroad for eyelid surgery, as well as for surgeons or academic societies seeking to increase social media presence or patient-directed educational content via social media engagement.

## Methods

### Ethical Considerations

This cross-sectional digital epidemiology study followed the World Medical Association’s ethical principles for medical research involving human participants outlined in the Declaration of Helsinki as amended in 2013. Institutional Review Board approval was not required as publicly available data were used. No identifiable patient information was involved in this study, and no compensation to participants took place. These methods were adapted from previous publications [6,13,15,16].

### Google Trends

In order to ensure that search terms accurately represent the intent of Americans searching the topic of aesthetic eyelid surgery abroad in an international destination, the Google Trends query was designed in the following way. Google Trends relative search volume (RSV) for US interest in aesthetic eyelid surgery abroad from April 1, 2013, to March 31, 2023, was collected on April 1, 2024. The query output is search volume as arbitrary values ranging from 0 to 100 for each query (maximum 5 search terms at a time) and referenced to the highest peak search popularity (set at 100) for the given terms, region, and time. As such, a standard RSV reference range was implemented across all 25 search terms by using a bracket type elimination where all queries were tested until the maximum was identified (Korea), and therefore Korea was always included as the most popular peak term (100) within each set of 5-term queries to ensure that all RSV values were relative to the same reference point. The location of origin of the Google searches was limited to the US geographic filter, and the “Cosmetic Surgery” category filter was applied to limit the output to aesthetic-surgery–related searches. A variety of search term combinations were generated and tested through trial and error until the term combinations with the greatest output were identified in order to minimize cells with low data counts. As a result, “eyelid nation” was found to produce the most data for temporal country analysis, while “blepharoplasty” produced the most data for geographic analysis in the United States. Quotation marks were not included in any queries.

A total of 25 destination countries were included according to the International Society of Aesthetic Plastic Surgery (ISAPS) Global Survey 2021 most popular cosmetic surgery destinations [5]. As such, the following twenty-five search terms were applied: (1) eyelid Argentina, (2) eyelid Australia, (3) eyelid Brazil, (4) eyelid Canada, (5) eyelid China, (6) eyelid Colombia, (7) eyelid France, (8) eyelid Germany, (9) eyelid Greece, (10) eyelid India, (11) eyelid Italy, (12) eyelid Japan, (13) eyelid Korea, (14) eyelid Mexico, (15) eyelid Netherlands, (16) eyelid Portugal, (17) eyelid Romania, (18) eyelid Russia, (19) eyelid Saudi Arabia, (20) eyelid Spain, (21) eyelid Taiwan, (22) eyelid Thailand, (23) eyelid Turkey, (24) eyelid United Kingdom, and (25) eyelid Venezuela. Control terms as a proxy for levels of general internet traffic in the same time period included “weather,” “sports,” “google,” and “news.” RSV data for aesthetic eyelid surgery overall in each US state during this period were extracted using the search topic “blepharoplasty.”

To evaluate the most popular destination countries based on average search interest, the average search volume from 2013‐2023 for each country were compared. To analyze the changes in search interest over time, the following calculations were performed to normalize aggregate RSV data on a standard scale of 0 to 100. First, the search volume of all international countries was summed together for each month from April 2013 to March 2023 to be termed eyelid surgery abroad. The same was done for the control terms. The monthly aggregate data for each category were then normalized to be set on a scale of 0 to 100 in the same fashion performed by Google Trends, which sets the maximum value of each category to 100. Normalization is achieved by dividing each monthly data point by the maximum value of the category, and then multiplying by 100. Next, the normalized monthly data for eyelid surgery abroad, blepharoplasty overall, and control were averaged into seasonal data based on the following seasons: Winter (December, January, and February), Spring (March, April, and May), Summer (June, July, and August), and Fall (September, October, and November).

### Social Media

After identifying America’s most desired eyelid cosmetic tourism destinations as above, the most popular international aesthetic eyelid surgeons on social media within those countries were identified in the following manner. The social media platform, Instagram, was chosen as it has been previously demonstrated that a majority of patients prefer Instagram for finding an oculofacial plastic surgeon [10].

Instagram was manually queried on May 1, 2024, for top eyelid surgery–related posts using the following search strategy based on previous methodologies [13]. Both hashtags and topic searches, including a variety of medical and layperson terminology, were used for the United States and each of the top 5 countries of highest demand extracted from the Google Trends analysis above (South Korea, Mexico, Canada, Turkey, and China). Through trial and error testing, additional similar hashtags or topic searches were added as needed to generate a minimum of 10 top posts for each country of interest. Instagram uses algorithm-based sorting to show a grid of the 9 top posts for a query based on factors like engagement (likes, comments, and shares), relevance to the query terms, and recency [13,17]. Search terms included the following: (1) #blepharoplastykorea, (2) #eyelidsurgerykorea, (3) #koreaeyelidsurgery, (4) eyelid surgery Korea, (5) #blepharoplastymexico, (6) #eyelidsurgerymexico, (7) #blepharoplastytijuana, (8) #eyelidsurgerytijuana, (9) eyelid surgery Mexico, (10) #eyelidsurgerycanada, (11) eyelid surgery Canada, (12) #blepharoplastyturkey, (13) #eyelidsurgeryturkey, (14) #eyelidsurgeryistanbul, (15) #blepharoplastychina, (16) #eyelidsurgerychina, (17) eyelid surgery China, (18) #blepharoplasty (representing United States), (19) #eyelidsurgery (representing United States), and (20) #eyelidlift (representing United States).

Content analysis was performed via a human annotator evaluating each top post for topic outputs to ensure they aligned with the theme identified for that topic. Inclusion criteria for content included posts relevant to eyelid surgery posted by an eyelid surgeon (ie, promoting services or medical tourism, photographs or videos in the operating room, before and after photos, anatomical diagrams, and patient-directed educational material). Duplicate posts, posts not relevant to eyelid surgery, or posts with an inability to access the appropriate information (blocked access or insufficient surgeon details) were excluded from the analysis. After removing duplicates, 55 unique posts were analyzed. The Instagram profile for each post was then mined for the surgeon’s website, or if not listed then the surgeon’s practice website was identified using search engine informatics querying the surgeon’s name on Google. Data extracted from each surgeon’s professional website included procedures, residency, fellowship, country of practice, professional medical society affiliations, warranties, and medical tourism packages. Data collected for membership to professional organizations were verified on the corresponding academic society website member lists and included American Society of Ophthalmic Plastic & Reconstructive Surgery (ASOPRS); Canadian Society of Oculoplastic Surgeons (CSOPS); American Academy of Facial Plastic & Reconstructive Surgery (AAFPRS); Fellow of the American College of Surgeons; American Society of Plastic Surgeons; European Board of Plastic Reconstructive & Aesthetic Surgery; Fellow of the Royal College of Surgeons of Canada; ISAPS; Oriental Society of Aesthetic Plastic Surgery; International Confederation for Plastic, Reconstructive & Aesthetic Surgery; Korean Academy of Facial Plastic and Reconstructive Surgery; Korean Society of Plastic and Reconstructive Surgeons; Asociación Mexicana de Cirugía Plástica Estética y Reconstructiva; and Canadian Society of Plastic Surgeons.

### Statistical Analysis

Statistical analysis used Microsoft Excel Version 16.66.1 (Microsoft Corporation) and GraphPad Prism QuickCalcs (GraphPad Software). Descriptive statistics were used to analyze the search interest volume over time, as well as the demographics of the international eyelid surgeons. Chi-square tests compared proportions for categorical variables between international and US eyelid surgeons.

## Results

### Google Trends

**Figure 1.**
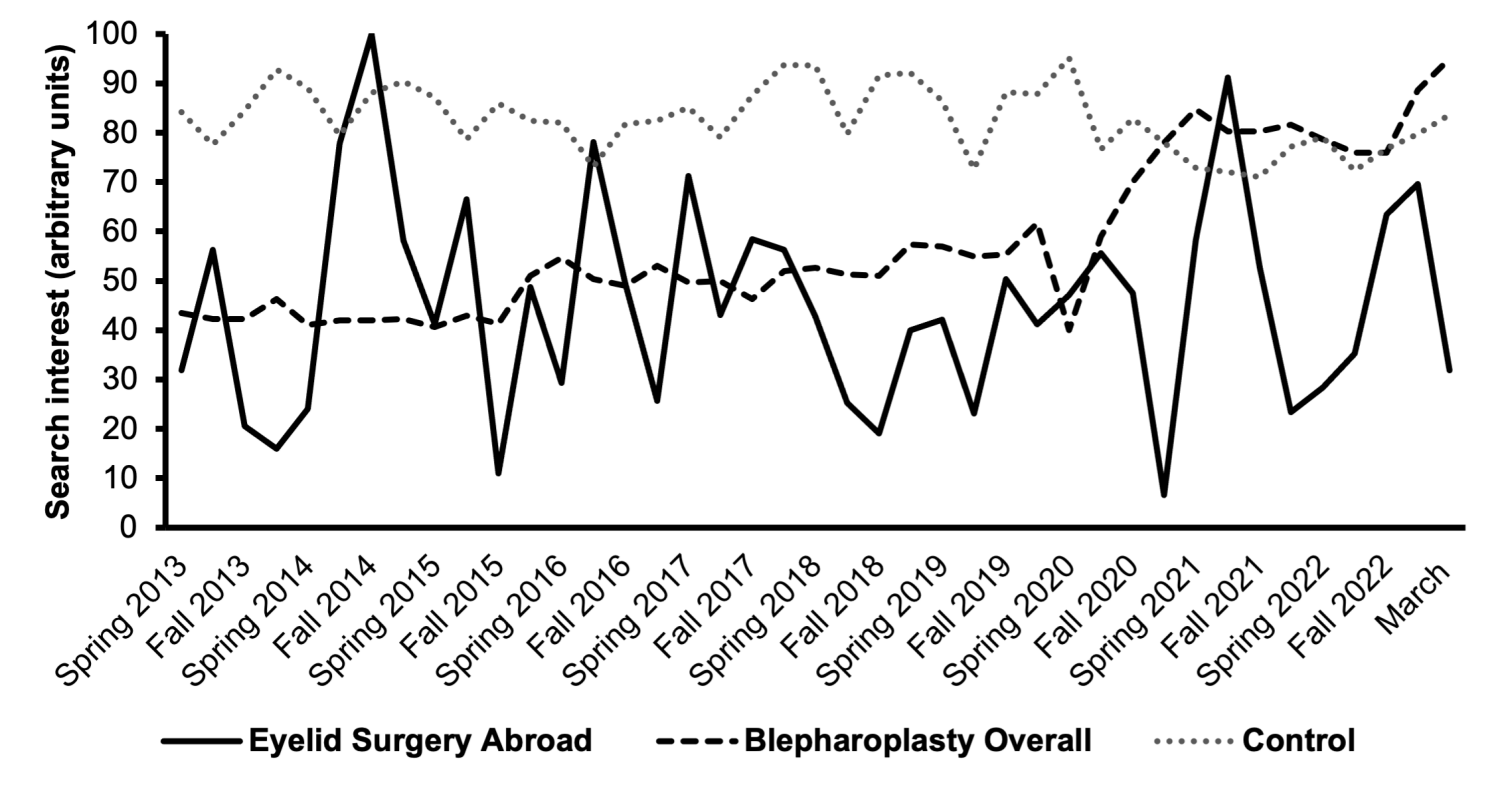
Google Trends search interest in cosmetic tourism for eyelid surgery**.** Despite periodic fluctuations, American Google Trends search interest in cosmetic tourism for eyelid surgery remained stagnant (0% growth) from 2013 to 2023, while overall interest in blepharoplasty rose 118.4%.

Between 2013 and 2023, the top 5 destinations for Americans seeking eyelid surgery abroad were South Korea, Mexico, Canada, Turkey, and China (Multimedia Appendix 1). Despite 118% growth in blepharoplasty searches, interest in eyelid surgery abroad remained steady (Figure 1). The notable growth occurred recently, averaging 30.61 (SD 2.49) increased RSV from June 2020 to March 2023 compared with previous years from April 2013 to May 2020 (95% CI 25.73-35.49 RSV; *P*<.001). US states with the highest blepharoplasty RSV were Florida, California, Hawaii, Nevada, and New York. While complete state-level data for interest in eyelid surgery in all the destination countries were unavailable, California led the United States in searches for eyelid surgery in South Korea.

### Social Media

In total, 55 top Instagram posts were included from aesthetic eyelid surgeons across ophthalmology, Otolaryngology-Head and Neck Surgery (OHNS), and plastic surgery. Within each discipline, qualifying fellowship training specializing in aesthetic eyelid surgery was examined. For ophthalmology, this included ASOPRS fellowship, CSOPS fellowship, or an unspecified OPS fellowship; for OHNS, AAFPRS facial plastic and reconstructive surgery fellowship or other unspecified or international facial plastic and reconstructive surgery fellowships; for plastic surgery, aesthetic plastic surgery fellowship.

When combining both the United States and international eyelid surgeons within each discipline, ophthalmology (7/10, 70%) and OHNS (9/13, 69%) were far more likely to have had fellowship training that included aesthetic eyelid surgery than plastic surgeons (1/32, 3%; *P*<.001) (Table 1). Of the plastic surgeons performing aesthetic eyelid surgery, 19% (4 abroad and 2 in the United States) were initially trained as general surgeons and subsequently obtained further training in general plastic surgery.

**Table 1.** Summary of training and professional society affiliations of most popular aesthetic eyelid surgeons on Instagram.

Specialty	Korea, n	Mexico, n	Canada, n	Turkey, n	China, n	Intl^a^, n (%)	United States, n (%)	*P* value
Ophthalmology	0	0	3	3	0	6 (14)	4 (36)	.08
ASOPRS^b^ fellowship	0	0	0	0	0	0 (0)	3 (27)	<.001
CSOPS^c^ fellowship	0	0	1	0	0	1 (2)	0 (0)	.61
Other OPS^d^ fellowship	0	0	2	0	0	2 (5)	1 (2)	.55
Total OPS fellowship	0	0	3	0	0	3 (7)	4 (36)	.009
OHNS^e^	1	1	2	4	0	8 (18)	5 (46)	.06
AAFPRS^f^ fellowship	0	0	1	0	0	1 (2)	5 (46)	<.001
Other FPS^g^ fellowship	0	0	1	2	0	3 (7)	0 (0)	.37
Total FPS fellowship	0	0	2	2	0	4 (9)	5 (46)	.004
Plastic surgery	9	10	5	5	1	30 (68)	2 (18)	.003
Aesthetic fellowship	0	0	0	1	0	1 (2)	0 (0)	.61
Total fellowship overall	0	0	5	3	0	8 (18)	9 (82)	<.001
Professional society^h^	5	8	7	2	0	22 (50)	11 (100)	.002
Total	10	11	10	12	1	44	11	—^i^

a Intl: international*.*

b ASOPRS: American Society of Ophthalmic Plastic & Reconstructive Surgery.

c CSOPS: Canadian Society of Oculoplastic Surgeons.

d OPS: oculofacial plastic surgery.

e OHNS: Otolaryngology–Head and Neck Surgery.

f AAFPRS: American Academy of Facial Plastic and Reconstructive Surgery.

g FPS: Facial Plastics.

h Professional Organizations included American Academy of Facial Plastic & Reconstructive Surgery (AAFPRS); American Society of Ophthalmic Plastic & Reconstructive Surgery (ASOPRS); Canadian Society of Oculoplastic Surgeons (CSOPS); Fellow of the American College of Surgeons (FACS); American Society of Plastic Surgeons (ASAPS); European Board of Plastic Reconstructive & Aesthetic Surgery (EBOPRAS); Fellow of the Royal College of Surgeons of Canada (FRCSC); International Society of Aesthetic Plastic Surgery (ISAPS); Oriental Society of Aesthetic Plastic Surgery (OSAPS); International Confederation for Plastic, Reconstructive & Aesthetic Surgery (IPRAS), Korean Academy of Facial Plastic and Reconstructive Surgery (KAFPRS), Korean Society of Plastic and Reconstructive Surgeons (KSPRS), Asociación Mexicana de Cirugía Plástica Estética y Reconstructiva (AMCPER), and Canadian Society of Plastic Surgeons (CSPS).

i Not applicable.

Looking specifically at the United States versus international surgeons, the US surgeons more frequently completed fellowships that encompassed aesthetic eyelid surgery–specific training compared with international surgeons (9/11, 82% vs 10/44, 23%; *P*<.001) (Table 1). Within ophthalmology, US aesthetic eyelid surgeons more often had OPS training compared with international surgeons (4/11, 36% vs 3/44, 7%; *P*=.009). Within OHNS, US surgeons more often had facial plastic surgery (FPS) fellowship training compared with international surgeons (5/11, 46% vs 4/44, 9%; *P*=.004), and especially had more AAFPRS fellowship training (5/11, 46% vs 1/44, 2%; *P*<.001). Interestingly, 100% (9/9) of the US ophthalmologists and US OHNS had done an OPS or FPS fellowship, respectively, compared with only 50% (3/6) of the international ophthalmologists and 50% (4/8) of the international OHNS, although this was not statistically significant (*P*=.06 and *P*=.09, respectively). General plastic surgery accounted for the majority of training for the most popular international eyelid surgeons, contrasting with a low proportion in the United States (30/44, 68% vs 2/11, 18%; *P*=.003). These plastic surgeons rarely had aesthetic plastic surgery fellowship training both abroad and in the US (1/30, 2% vs 0/2, 0%; *P*=.61).

Aesthetic eyelid surgeons in the United States were more likely to have active membership in recognized professional medical societies than their international counterparts (11/11, 100% vs 22/44, 50%; *P*=.002). A summary of these professional societies is listed in Table 1.

Among aesthetic eyelid surgeons abroad, 27% (12/44) offered medical tourism travel packages, while only 5% (2/44) mentioned warranty systems for the financial handling of revisional surgery on their websites.

## Discussion

### Principal Findings

This infodemiology study indicates Americans primarily seek aesthetic eyelid surgery abroad in Asia and Europe (South Korea, Turkey, and China) as well as the US neighboring countries (Mexico and Canada). These findings align with existing plastic surgery literature. ISAPS 2021 ranked Turkey, Colombia, Mexico, Thailand, and Spain as top aesthetic surgery destinations [5]. Previous studies suggest these trends may reflect proximity (eg, Mexico and Canada in this United States–based study, or Spain in the largely European-based ISAPS study) and niche surgical offerings (eg, Thailand for gender reassignment or Colombia for breast, body, and extremity) [5,18]. South Korea’s popularity for eyelid surgery among Americans may be linked to the nation’s commonly sought-after idealized appearance referred to as the Korean look achieved through a distinct set of facial cosmetic procedures defined in South Korea and popularized by global exportation of South Korean popular culture [19]. These aesthetic surgeries produce a desired appearance for the East Asian face, focused on widening the eyes, narrowing the cheekbones and jawbones, and augmenting the nose tip. In this study, most interest in South Korean eyelid surgery originated from California, home to half a million of the United States’ 1.7 million Korean Americans, suggesting a shared cultural desire for the Korean look [20].

This study demonstrates overall American interest in blepharoplasty doubled in the last decade with most growth occurring after the United States lifted COVID-19 restrictions in May 2020. However, this has not translated to a greater preference for international destinations for aesthetic eyelid surgery, possibly attributed to reopening patterns after COVID-19 travel restrictions. These findings expand on previous studies that found declines in domestic interest in oculofacial plastic, facial plastic, or general plastic surgery and a temporary rise in interest abroad during the pandemic [3,6]. By 2021, the United States rebounded to 85.7% of prepandemic cosmetic surgery volume, making the US surgeons more accessible than the surgeons abroad postrestrictions [5].

The US aesthetic eyelid surgeons popular on social media more often had fellowship training specializing in blepharoplasty techniques, including OPS fellowship or FPS fellowship. They were also more likely to hold membership in a professional medical society than their international counterparts. A recent study on problematic Instagram medical marketing demonstrated a significant number of physicians advertising as cosmetic surgeons without appropriate plastic surgery credentials [13]. Ideally, consumers should be aware of differences in specialty training and qualifications when selecting an aesthetic eyelid surgeon.

A quarter of international eyelid surgeons offered medical tourism vacation packages, involving substantial upfront financial commitments that may pressure patients to proceed with surgery. Cosmetic tourists may also encounter a lack of language concordance, psychological stress from traveling and recovering alone, inadequate follow-up, limited legal recourse, and surgical complications including perioperative fatalities [1-3]. Of note, few international aesthetic eyelid surgeons were observed to offer a warranty. It is customary in the United States for eyelid surgery practices to have a revisions policy addressing the possible event of additional corrective procedures; while this may not necessarily be displayed on the surgeon’s website, it is an important aspect of the surgical discussion and consent process. For international surgeons, on the other hand, displaying the warranty policy on the surgeon’s website may be more essential as reassurance to the patient. Of note, the Centers for Disease Control and Prevention recommends that cosmetic tourism patients should be informed of their rights and legal recourse before agreeing to travel outside the United States for medical care because of the high costs of complications and limited ability for legal action [21].

Aesthetic surgery remains a key contributor in the US $27.8 billion global medical tourism industry, earning high satisfaction ratings in some populations [22]. Future investigations should explore cost, desire for local expertise, and perceptions of international experience to better appreciate the American interest in aesthetic eyelid surgery abroad. In addition, an examination of a particular destination’s aesthetic OPS niche may shed further light on cosmetic preferences and tourism patterns. Finally, an understanding of how social media’s relative lack of international aesthetic eyelid surgeons with fellowship training that includes blepharoplasty techniques correlates to surgical outcomes and patient satisfaction would provide further insight for Americans seeking cosmetic tourism abroad. Such information may also help guide professional academic society resource allocation and social media engagement.

### Limitations

Google Trends lacks patient demographics and outcomes, can be influenced by media exposure or user manipulation, and may encompass users beyond those seeking surgery. Social media may portend representation gaps for less tech-savvy surgeons. Instagram restrictions limit data from China, precluding conclusions about the country. Surgeons’ websites may not disclose all details. While the Google and Instagram platforms are popular United States–based channels for health information–seeking, these may not represent the only relevant media platforms from an international standpoint. Relevant data for each country may not have been captured by the specific hashtags used; however, the presence of several duplicate posts among variations of hashtags and topic queries supports the appropriateness of these terms in highlighting the top content. Analysis of surgical qualifications is limited as different countries have inherently different training and licensing regulations, as well as cultural variations and legal requirements in how they may be advertised. The study is limited to internet search trends and social media trends, but the reasons behind these trends remain unclear. The search terms designed for geographic identification may not represent all queries for eyelid surgery abroad. Rate limiting on posts collected on Instagram may confound the output for all or a number of countries. In consistency with previous research, the sample of social media data was queried at one point in time to control for temporal variations in content engagement; however, this strategy may limit the generalizability of the content to other seasons of the year. Given these limitations, the study should be received as exploratory, laying a preliminary foundation of novel insight in the unexplored area of cosmetic tourism for eyelid surgery.

### Conclusions

This study used Google Trends and social media to identify preferred international destinations among US travelers seeking cosmetic eyelid surgery and examined specialty training of the most sought-after aesthetic surgeons in those countries trending on social media. It highlights the shortage of international surgeons with aesthetic eyelid surgery–specific fellowship training and membership in a recognized professional medical society. Further research is necessary to evaluate how these trends correlate with demographics, surgical outcomes, and niche surgical offerings.

## Supplementary material

10.2196/64639 Multimedia Appendix 1 Top 5 destinations Americans seek for eyelid surgery abroad. The most sought-after destinations for cosmetic eyelid surgery abroad were compared based on US consumers’ Google searches for eyelid surgery internationally in 25 countries from 2013 to 2023.

## Abbreviations

AAFPRS: American Academy of Facial Plastic & Reconstructive Surgery
ASOPRS: American Society of Ophthalmic Plastic & Reconstructive Surgery
CSOPS: Canadian Society of Oculoplastic Surgeons
FPS: facial plastic surgery
ISAPS: International Society of Aesthetic Plastic Surgery
OHNS: Otolaryngology-Head and Neck Surgery
OPS: oculofacial plastic surgery
RSV: relative search volume

## References

[1] Raggio BS, Brody-Camp SA, Jawad BA, Winters RD, Aslam R (2020). Complications associated with medical tourism for facial rejuvenation: a systematic review. Aesthetic Plast Surg.

[2] Pereira RT, Malone CM, Flaherty GT (2018). Aesthetic journeys: a review of cosmetic surgery tourism. J Travel Med.

[3] Murphy D, Lane-O’Neill B, Dempsey MP (2022). COVID-19 and cosmetic tourism: a Google Trends analysis of public interests and the experience from a tertiary plastic surgery centre. J Plast Reconstr Aesthet Surg.

[4] Wilde C, Ross AR, Maharajan S (2018). Health tourism and the need for occasional strong paternalism: complications and management of cosmetic anterior chamber iris implantation. Eye (Lond).

[5] (2021). ISAPS International Survey on Aesthetic/Cosmetic Procedures performed in 2021. https://www.isaps.org/media/vdpdanke/isaps-global-survey_2021.pdf.

[6] Azzam DB, Cypen SG, Tao JP (2021). Oculofacial plastic surgery-related online search trends including the impact of the COVID-19 pandemic. Orbit.

[7] Tijerina JD, Morrison SD, Nolan IT, Vail DG, Nazerali R, Lee GK (2019). Google Trends as a tool for evaluating public interest in facial cosmetic procedures. Aesthet Surg J.

[8] Teixeira JC, Morrison SD, Brandstetter KA, Nuara MJ (2020). Is there an increasing interest in facial feminization surgery? A search trends analysis. J Craniofac Surg.

[9] Cheng T, Wang F, Barmettler A (2022). #Oculoplastics: an analysis of TikTok’s top oculoplastics content. Ophthalmic Plast Reconstr Surg.

[10] Alshaalan HS, AlTamimi LA, Alshayie RA, Alsuhaibani AH (2021). The impact of social media accounts on periocular cosmetic surgeries. Saudi J Ophthalmol.

[11] Tingley J, Allen RC, Barmettler A (2022). #OculoplasticsandSocialMedia: a review of social media in oculoplastics and relevant subspecialties. Orbit.

[12] Park SSE, Akella SS, Moon JY (2020). Building your brand: analysis of successful oculoplastic surgeons on social media. Ophthalmic Plast Reconstr Surg.

[13] Dorfman RG, Vaca EE, Mahmood E, Fine NA, Schierle CF (2018). Plastic surgery-related hashtag utilization on Instagram: implications for education and marketing. Aesthet Surg J.

[14] Gunawardene AN, Suraneni S, Rohowetz LJ, Sridhar J (2025). Characteristics and medical accuracy of online discussions of retinal conditions on a social media platform. J Vitreoretin Dis.

[15] Azzam DB, Nag N, Tran J (2021). A novel epidemiological approach to geographically mapping population dry eye disease in the United States through Google Trends. Cornea.

[16] Nuti SV, Wayda B, Ranasinghe I (2014). The use of Google Trends in health care research: a systematic review. PLoS ONE.

[17] (2025). How posts appear on Instagram hashtag search results. Instagram.

[18] Franzblau LE, Chung KC (2013). Impact of medical tourism on cosmetic surgery in the United States. Plast Reconstr Surg Glob Open.

[19] Holliday R, Cheung O, Cho JH, Bell D (2017). Trading faces: The ‘Korean Look’ and medical nationalism in South Korean cosmetic surgery tourism. Asia Pac Viewp.

[20] Ivey SL, Kim H, Yoo E (2019). Health and healthcare needs of Koreans in San Francisco Bay Area: The Korean Needs Assessment (KoNA) project. J Immigr Minor Health.

[21] (2023). Medical tourism CDC yellow book 2024. US Centers For Disease Control and Prevention.

[22] Campbell A, Restrepo C, Navas G (2020). Patient satisfaction with medical tourism: a review of 460 international plastic surgery patients in Colombia. Plast Reconstr Surg Glob Open.

