# Supplementary figures and images for: Global Surveillance of Public Interest in Cosmetic Tourism for Aesthetic Eyelid Surgery Abroad: Cross-Sectional Infodemiology Investigation of Internet Search Trends and Social Media Content
Source: JMIR Infodemiology. 2025 Jun 2;5:e64639. doi: 10.2196/64639 (PMC12148249; doi:10.2196/64639)

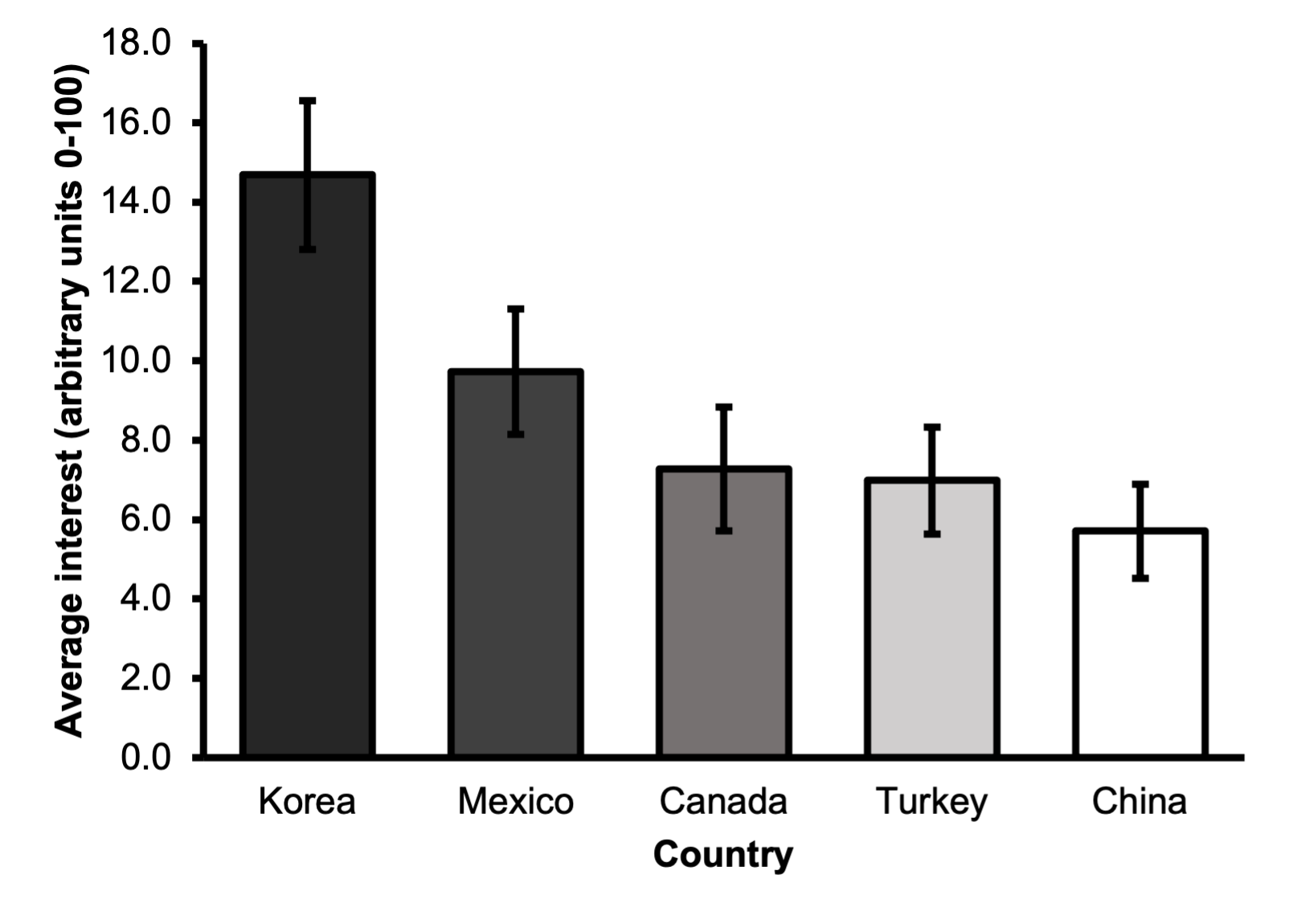

Supplement: Multimedia Appendix 1 [file infodemiology-v5-e64639-s001.png]
